# Characterization of the Culturable Sporobiota of Spanish Olive Groves and Its Tolerance toward Environmental Challenges

**DOI:** 10.1128/spectrum.04013-22

**Published:** 2023-01-31

**Authors:** Julia Manetsberger, Natacha Caballero Gómez, Nabil Benomar, Graham Christie, Hikmate Abriouel

**Affiliations:** a Área de Microbiología, Departamento de Ciencias de la Salud, Facultad de Ciencias Experimentales, Universidad de Jaén, Jaén, Spain; b Department of Chemical Engineering and Biotechnology, University of Cambridge, Cambridge, United Kingdom; Beijing Forestry University

**Keywords:** sporobiota, *Bacillus* spp., soil microbiome, olive agriculture, environmental resistance

## Abstract

Olive agriculture presents an integral economic and social pillar of the Mediterranean region with 95% of the world’s olive tree population concentrated in this area. A diverse ecosystem consisting of fungi, archaea, viruses, protozoa, and microbial communities—the soil microbiome—plays a central role in maintaining healthy soils while keeping up productivity. Spore-forming organisms (i.e., the sporobiota) have been identified as one of the predominant communities of the soil microbiome and are known for the wide variety of antimicrobial properties and extraordinary resistance. Hence, the aim of this work was to determine the culturable sporobiota of Spanish olive orchards and characterize its phenotypic properties toward common environmental challenges. A collection of 417 heat-resistant bacteria were isolated from five Spanish olive orchards. This collective was termed the “olive sporobiota.” Rep-PCR clustering of representative isolates revealed that they all belonged to the group of Bacillus spp., or closely related species, showing a great variety of species and strains. Representative isolates showed susceptibility to common antibiotics, as well as good resistance to heavy metal exposure, with an order of metal tolerance determined as iron > copper > nickel > manganese > zinc > cadmium. Finally, we showed that the application of mineral fertilizer can in several cases enhance bacterial growth and thus potentially increase the relative proportion of the sporobiota in the olive grove ecosystem. In summary, the identification of the culturable olive sporobiota increases our understanding of the microbial diversity in Spanish olive groves, while tolerance and resistance profiles provide important insights into the phenotypic characteristics of the microbial community.

**IMPORTANCE** Microbial communities are a key component of healthy soils. Spore-forming microorganisms represent a large fraction of this community—termed the “sporobiota”—and play a central role in creating a conducive environment for plant growth and food production. In addition, given their unique features, such as extraordinary stability and antimicrobial properties, members of the sporobiota present interesting candidates for biotechnological applications, such as sustainable plant protection products or in a clinical setting. For this, however, more information is needed on the spore-forming community of agricultural installations, ultimately promoting a transition toward a more sustainable agriculture.

## INTRODUCTION

The olive is one of the most precious crops produced in the Mediterranean region, concentrating 95% of the world’s olive trees and with Spain as the largest olive oil producer worldwide (International Olive Council, 13.1.2022, https://www.internationaloliveoil.org/the-world-of-olive-oil/). This crop, like any other commercial crop, is inherently susceptible to known and emerging plant pests and infectious diseases caused by bacteria, fungi, and viruses. Hence, there is a dire need for novel biological plant protection products that fulfill current policy requirements, such as the highly ambitious targets set in the European Commission’s “Green Deal” (https://ec.europa.eu/clima/eu-action/european-green-deal_en), while at the same time ensuring to keep up production and quality levels (1).

Healthy soils are necessary for efficient and healthy food production, maintaining biodiversity, reversing biodiversity loss, and fighting climate change. In this regard, an essential part of healthy soils is its diverse ecosystem, consisting of fungi, archaea, viruses, protozoa, and microbial communities, collectively called the soil microbiome (2). Similar to the human microbiome, this collective fulfills crucial functions to keep the soil and plants in good condition, such as provision and cycling of nutrients or decomposition of organic matter (3, 4). Several classes of soil microbes have been identified to particularly sustain plant growth and have consequently been termed “plant growth-promoting bacteria” (PGPB) (5). The predominant PGPB include members of the genera Azospirillum, Azotobacter, Klebsiella, Enterobacter, Alcaligenes, Arthrobacter, Burkholderia, Pseudomonas, and Bacillus, with the latter two considered the predominant communities (6). Notably, Bacillus spp. represent up to 95% of Gram-positive populations and are one of the most widespread endophytic bacteria (7, 8).

Situated in the phylum *Bacillota*, the Gram-positive genus Bacillus encompasses a wide range of pathogenic and nonpathogenic species and subspecies, some of which enjoy GRAS (generally regarded as safe) status (9). Due to its extensive polyphyly, a reclassification of the genus Bacillus has recently been proposed, notably to recognize 17 Bacillus species into novel genera, while limiting the genus Bacillus strictly to subtilis and cereus clades (10). Members of the Bacillus genera (as traditionally defined) are further characterized by their ability to form endospores when encountering unfavorable environmental conditions, such as nutrient starvation (11). These microstructures are one of the most stable structures found in nature, allowing Bacillus spp. to resist harsh conditions and persist in the environment over extended periods of time (12). Consequently, populations of Bacillus spp. can colonize a large variety of niches and are omnipresent in air, water, soil, and food (13). The collective of Bacillus spp. and other (related) spore-forming bacteria has been termed the “sporobiota,” highlighting its importance and distinct position within the bacterial microbiome (14).

Due to their unique properties, representatives of the Bacillus genus have a large variety of medical and biotechnological applications, such as the production of biofuels, biopolymers, or bioactive molecules (15). In an agricultural setting, Bacillus spp. can notably promote plant growth, act as biofertilizer or biostimulator, and inhibit pathogen growth and have thus been recognized as one of the most promising sustainable biocontrol agents (8). These properties have been demonstrated in a variety of economically important crops, such as wheat, sunflower, and potatoes (16). In this regard, Bacillus-based biofertilizers can improve plant growth and yield through their ability to (i) increase nutrient availability, (ii) nitrogen (N_2_) fixation and phosphorus-solubilizing properties, and (iii) phytohormone/plant-growth regulator production (8). On the other hand, due to their ability to produce antimicrobial compounds, in combination with their low production cost and high stability through spore formation, Bacillus spp. have been used in the past as biocontrol agents and present promising biological alternatives to chemical pesticides. Notably, Bacillus thuringiensis is considered already today as one of the most successful and widely used biological plant protection products worldwide thanks to its ability to produce toxin crystals with insecticidal properties (17), but other species, for example B. subtilis or Bacillus amyloliquefaciens, have also been commercialized for their successful application as biocontrol agents in a variety of commercial crops (18–20). Furthermore, the ability to produce bacteriocins—ribosomally produced antimicrobial peptides—has shown particular potential for antimicrobial plant treatment and has thus received considerable attention (8, 21). In this regard, it has been suggested to particularly focus on soil indigenous species for the development of novel biological plant protection products, in order to ensure maximum efficiency of antimicrobial activity in combination with maximum survival of the biocontrol agent, while at the same time guaranteeing minimum alteration of natural soil conditions (8, 22).

Given the above, in this study, we set out to determine the culturable sporobiota of Spanish olive groves. We have determined the composition of the Bacillus spp. and related species community present in these agricultural soils, as well as their resistance properties toward environmental challenges faced in large-scale agriculture. These challenges include the exposure to heavy metals, fertilizers, and antibiotic compounds, notably with a view to developing novel biocontrol agents.

## RESULTS

### Sample collection and isolation of the culturable olive sporobiota.

To isolate Bacillus species and related spore formers from olive tree leaves and the surrounding soil, a total of five different olive orchards in Andalusia, Spain, were selected (Table 1). To specifically select spore-forming organisms, the sample was heated to 78°C for 20 min, effectively removing vegetative (heat-labile) cells, while allowing the survival of only heat-resistant organisms (e.g., endospores). In general, low numbers of bacteria were isolated from the samples, especially from exogenic and endophytic leaf samples. This was also highly dependent on the sample origin.

**TABLE 1:** Summary of sample origin and number of culturable olive sporobiota isolates obtained in this study

Location	Abbreviation	Tree variant	Collected samples	Total isolates	Clustered culturable isolates
Linares (Jaén)	LIN	Picual	18	146	102
		Arbequina	6		
Jimena (Jaén)	JP	Picual	2	18	18
Bedmar y Garcíez	BG	Picual	4	23	22
La Guardia de Jaén	LGJ	Picual	38	171	144
Málaga	MA	Aloreña	12	59	57
Total			80	417	343

### Identification of the culturable olive sporobiota of Spanish olive orchards.

**FIG 1:**
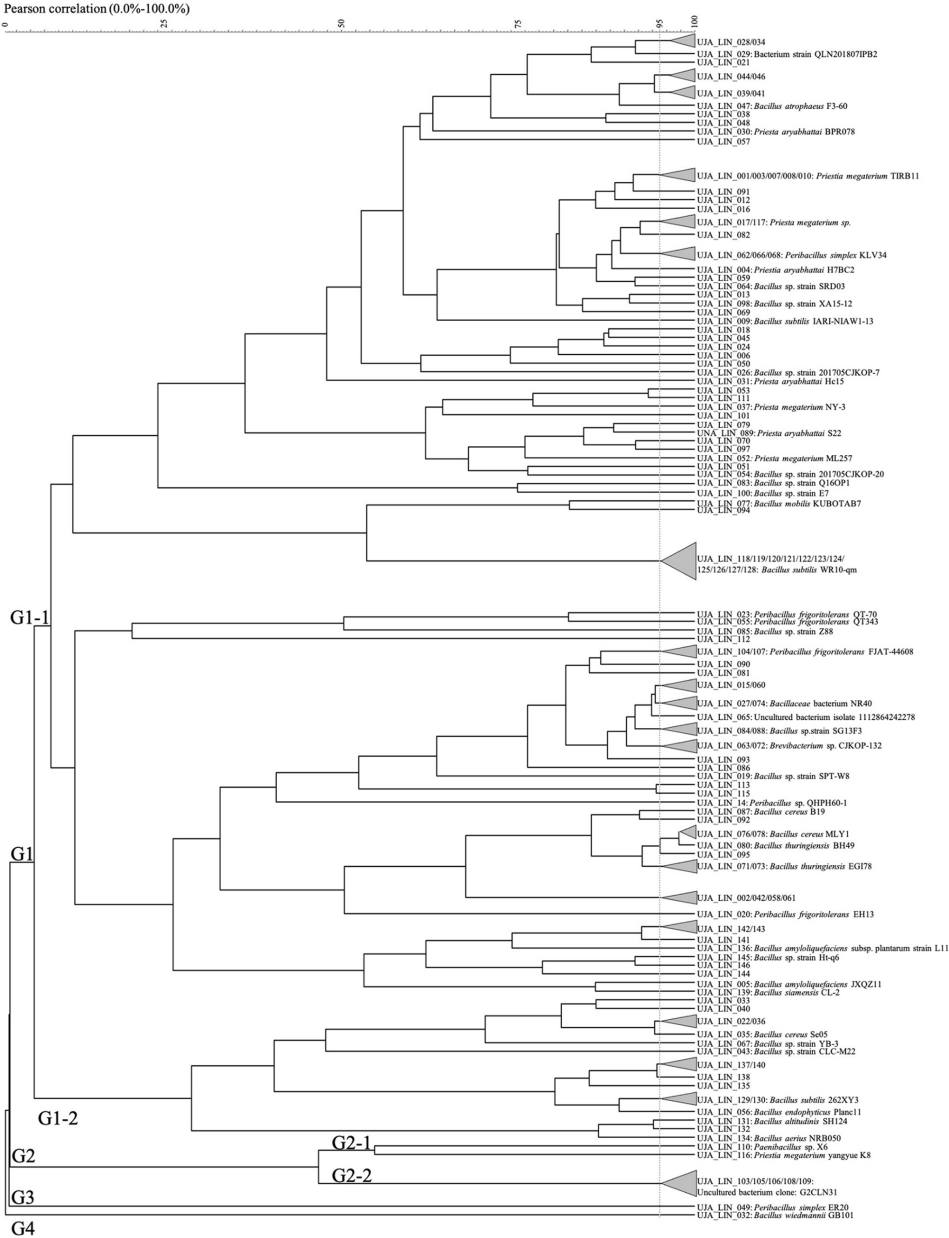
Dendrogram obtained from (GTG)_5_ fingerprints of the culturable olive sporobiota isolated from the Linares origin (LIN).

**FIG 2:**
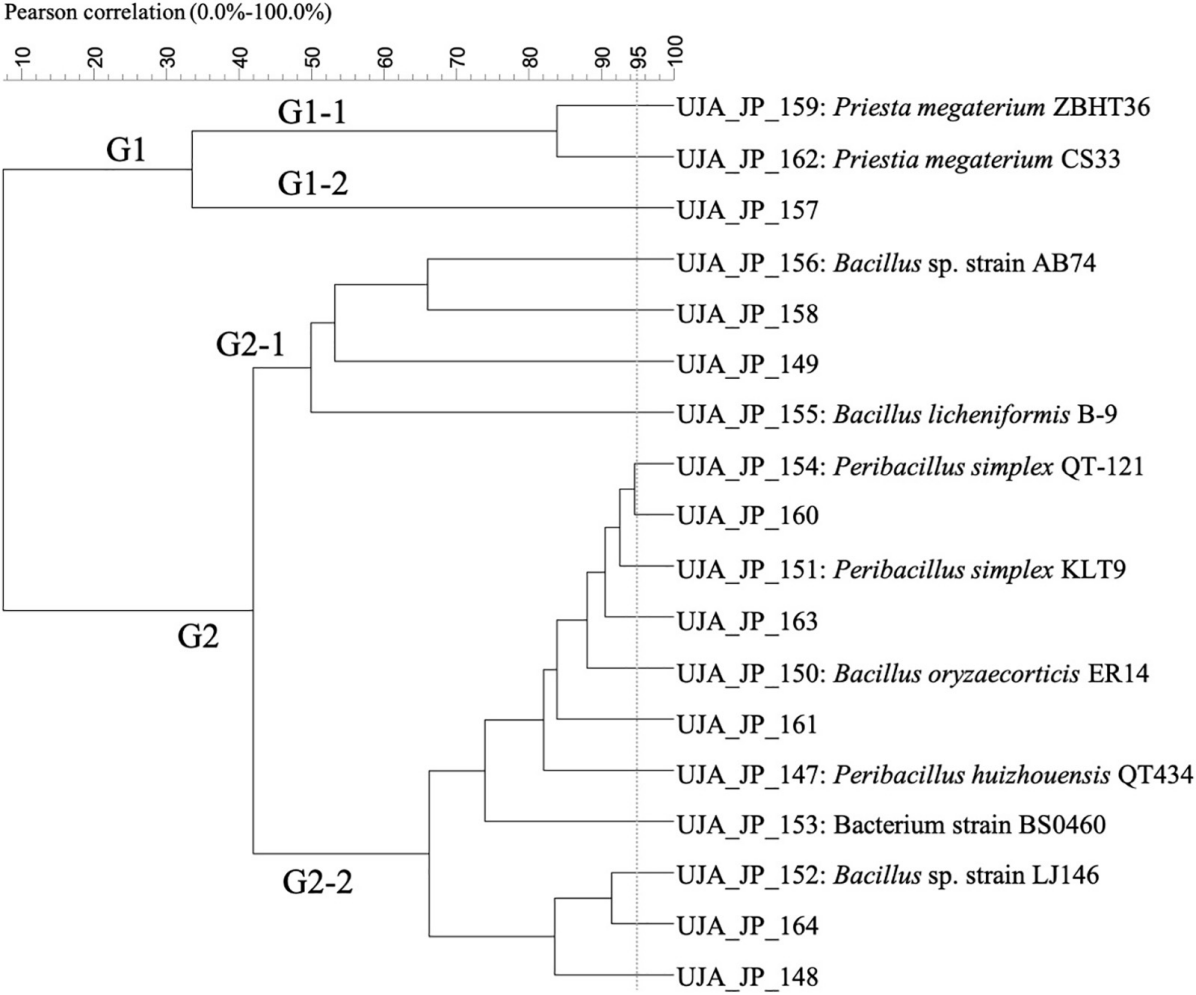
Dendrogram obtained from (GTG)_5_ fingerprints of the culturable olive sporobiota isolated from the Jimena origin (JP).

**FIG 3:**
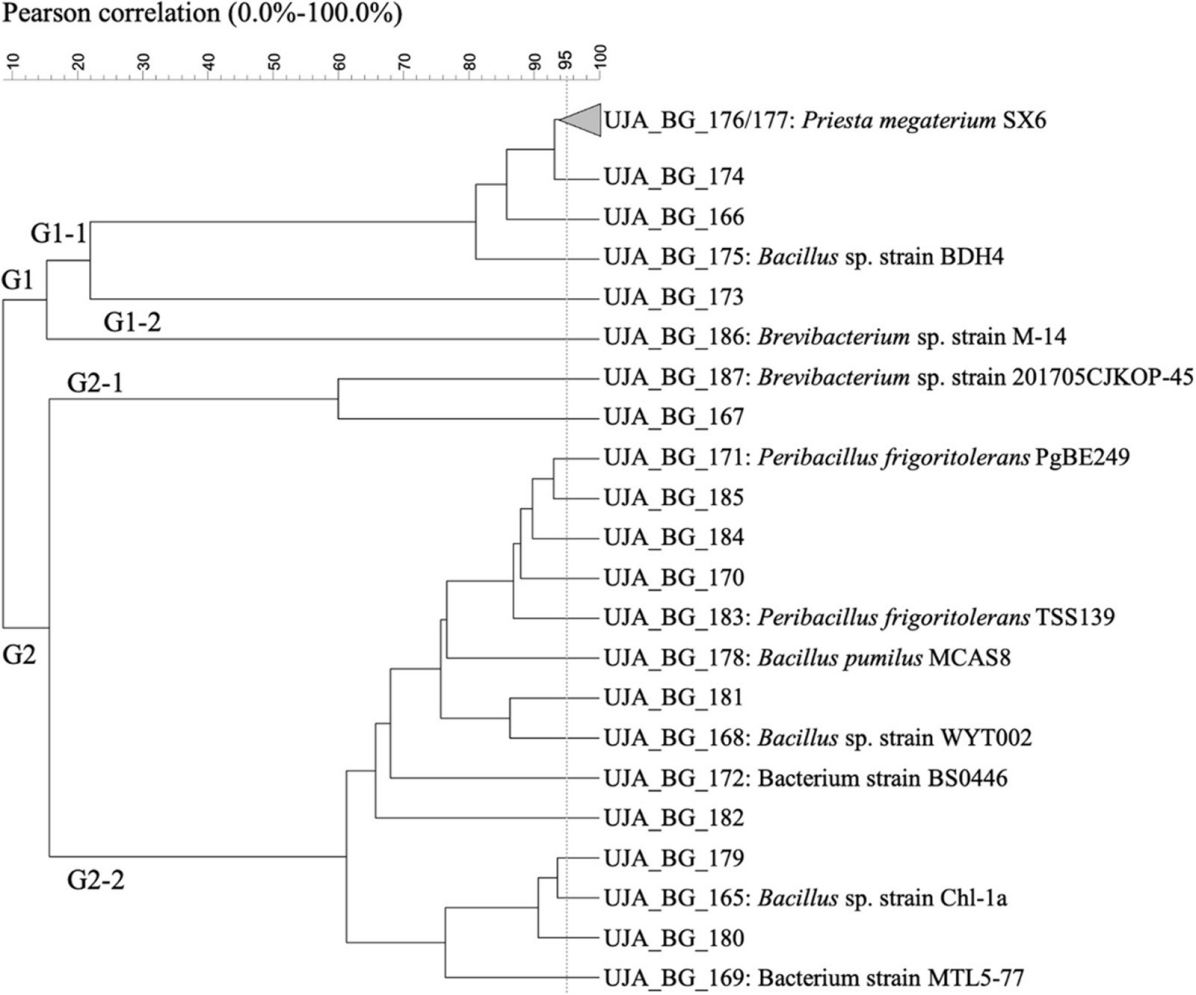
Dendrogram obtained from (GTG)_5_ fingerprints of the culturable olive sporobiota isolated from the Bedmar y Garcíez origin (BG).

**FIG 4:**
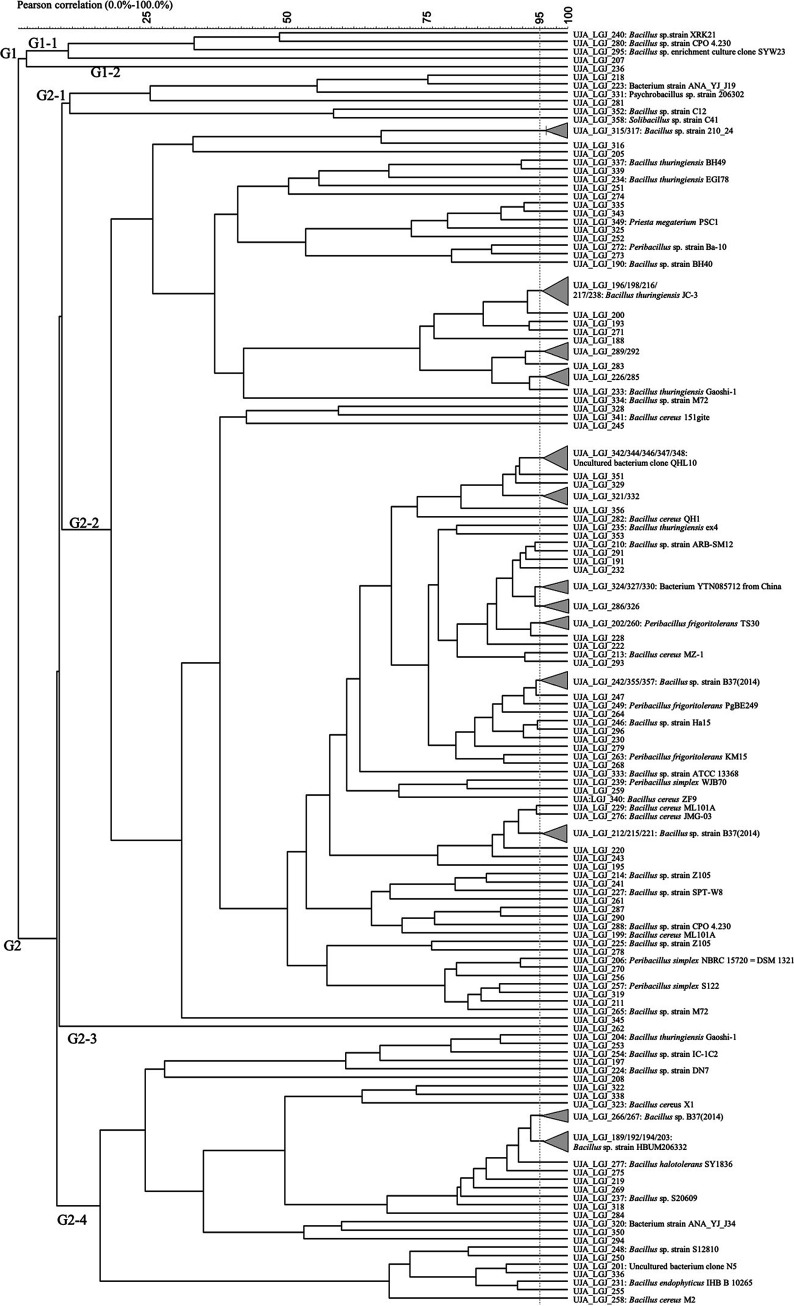
Dendrogram obtained from (GTG)_5_ fingerprints of the culturable olive sporobiota isolated from the La Guardia de Jaén origin (LGJ).

**FIG 5:**
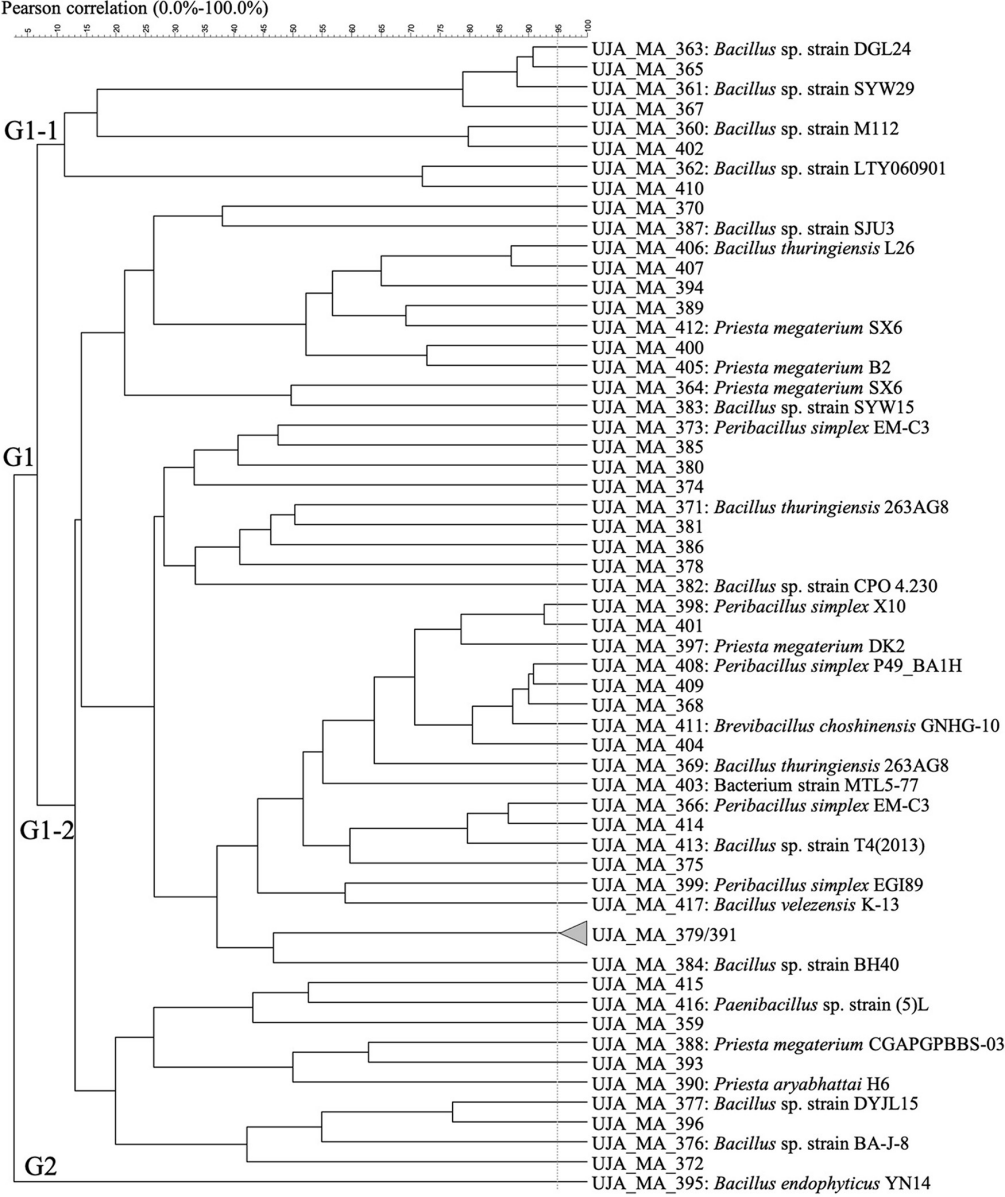
Dendrogram obtained from (GTG)_5_ fingerprints of the culturable olive sporobiota isolated from the Málaga origin (MA).

A collection of 417 isolates was obtained from five different origins, representative of the soil, leaf surface, and endophytic culturable spore-forming microbial population in Spanish olive orchards mainly isolated from La Guardia de Jaén (LGJ, 171 isolates) followed by Linares (LIN, 146 isolates), in addition to Málaga (MA, 59 isolates), Jimena (JP, 18 isolates), and Bedmar y Garcíez (BG, 23 isolates) (Table 1). Of these isolates, 10 appeared to be unculturable after the initial isolation step, leaving 407 candidates for further analysis. (GTG)_5_ Rep-PCR analysis allowed detection of clonal relationships between isolates. Results grouping isolates by origin are shown in Fig. 1 to 5. To identify isolates, 16S rRNA of representative species from each cluster were sequenced.

Cluster analysis of Rep-PCR patterns by origin reduced the 407 culturable isolates to 343 strains. In this regard, Rep-PCR analysis of isolates obtained from the LIN origin reduced isolates from 140 to 102 strains, revealing four main genomic groups (G1 to G4) (Fig. 1). G3 and G4 included one isolate each (UJA_LIN_049, Peribacillus simplex ER20; UJA_LIN_032, Bacillus wiedmannii GB101, respectively). G2 showed a further division into two subgroups: G2-1, comprised of two strains (UJA_LIN_110, Paenibacillus sp. X6; UJA_LIN_116, Priesta megaterium yangyue K8), and the isolated G2-2. The latter consisted of the clustered UJA_LIN_103/105/106/108/109 strain, identified as uncultured bacterium clone: G2CLN31. Most isolates belonged, however, to G1, which could be grouped into two subgroups (G1-1 and G1-2). Within these clusters, no predominant spore-forming species was detected, but they rather showed a high genetic diversity (Fig. 1; Table S1).

No reduction occurred after cluster analysis for JP isolates (18) (Fig. 2), which could be grouped into a small cluster G1 consisting of two isolates of the P. megaterium genus under the subgroup G1-1 (UJA_JP_159, P. megaterium ZBHTT36; UJA_JP_162, P. megaterium CS33) and the isolated G1-2 subgroup with strain UJA_JP_157, Priesta aryabhattai Y7. The remaining 15 isolates comprised the main cluster (G2). G2 notably showed a subdivision into two subgroups: G2-1 (4 isolates) and G2-2 (11 isolates), the latter of which contained various Peribacillus sp.

Rep-PCR profiling caused a reduction from 23 to 22 strains in isolates from BG orchards (Fig. 3). Analysis further yielded a clustering into 2 main clusters (G1 and G2). The smaller cluster (G1) consisted initially of 7 isolates, 2 of which were combined into 1 strain (UJA_BG_176 and UJA_BG_177) identified as P. megaterium SX6. The G1 cluster comprised 2 subgroups: G1-1 with 6 isolates and G1-2 with 1 isolate (UJA_BG_186, Brevibacterium sp. M-14). The larger cluster (G2) contained 16 isolates grouped into 2 subclusters: G2-1 with 2 representatives (UJA_BG_187, Brevibacterium sp. 201705; UJA_BG_167) and G2-2 with 14 isolates. As for previous origins, the BG orchard showed a large variety of spore-forming species with no particular distribution pattern.

The collection of 168 LGJ isolates was reduced to 144 after Rep-PCR cluster analysis (Fig. 4). Rep-PCR profiling notably revealed 2 main genomic groups: a minor group (G1) consisting of 5 isolates and a major group (G2). G1 could be grouped into the subcluster G1-1 with 4 isolates mostly identified as Bacillus sp. (without species identification) and the single isolate G1-2 (UJA_LGJ_236). G2 on the other hand showed a subdivision into 4 major subgroups (G2-1 to G2-4), demonstrating a large microbial diversity of the culturable olive sporobiota from this origin. G2-3 comprises only one strain UJA_LGJ_262, whereas the largest subgroup was G2-2 followed by G2-1 and G2-4 showing bacterial strain and genus heterogeneity. In this regard, a comparatively high number of Bacillus cereus isolates (7), as well as B. thuringiensis (5), were identified in G2-2.

Finally, the cluster analysis of Rep-PCR profiles of 58 isolates derived from the MA orchard yielded 57 strains. (Fig. 5) with an overall distribution into 2 clusters (G1 and G2). Here, G2 included only 1 isolate (UJA_MA_395, Bacillus endophyticus YN14), while cluster 1 comprised the great majority of species and strains isolated from this origin. Similar to clusters described from other origins, G1 showed various subdivisions, notably into G1-1 and G1-2, the latter of which split into further subgroups. 16S rRNA sequencing data for strains representative of the smaller G1-1 cluster (8 isolates) yielded mostly Bacillus sp. without further specifying the strains. On the other hand, cluster G1-2 included a large variety of species and strains.

As mentioned above, 16S rRNA sequencing and BLAST homology search tentatively identified representative species from each cluster. The results revealed a great diversity of bacterial isolates and homogeneity between origins (Table 2; Table S1). Of the total of 407 isolates, 158 were selected for strain identification based on their representative position in the Rep-PCR profile for each origin. Of these representative isolates, the most identified bacterium was Bacillus sp. (51 isolates), while the most prominent identified species were P. megaterium (14 isolates; previously Bacillus megaterium), closely followed by B. cereus (12 isolates) and P. simplex (12 isolates; previously Bacillus simplex), B. thuringiensis (11 isolates), Peribacillus frigoritolerans (9 isolates; previously Brevibacterium frigoritolerans), P. aryabhattai (5 isolates; previously Bacillus aryabhattai), Bacillus subtilis (3 isolates), B. endophyticus (3 isolates), B. amyloliquefaciens (2 isolates), Paenibacillus sp. (2 isolates), and Peribacillus sp. (2 isolates). The analysis further yielded one isolate each of Bacillaceae bacterium, Bacillus aerius, Bacillus altitudinis, Bacillus atrophaeus, Bacillus halotolerans, Bacillus licheniformis, Bacillus mobilis, Bacillus oryzaecorticis, Bacillus pumilis, Bacillus siamensis, Bacillus velezensis, Bacillus wiedmannii, Brevibacillus choshinensis, Peribacillus huizhouensis, Psychrobacillus sp., and Solibacillus sp. in addition to “bacterium strain” (9 isolates), Brevibacterium sp. (3 isolates), and “uncultured bacterium” (4 isolates).

**TABLE 2:** Representative isolates from the culturable olive sporobiota^*a*^

Bacterial species	16S rRNA gene identity (%)	Total	LIN	JP	BG	LGJ	MA
Bacillaceae bacterium	88.48	1	1	0	0	0	0
Bacillus aerius	98.20	1	1	0	0	0	0
Bacillus altitudinis	99.86	1	1	0	0	0	0
Bacillus amyloliquefaciens	99.79–99.93	2	2	0	0	0	0
Bacillus atrophaeus	97.76	1	1	0	0	0	0
Bacillus cereus	95.28–99.93	12	3	0	0	9	0
Bacillus endophyticus	99.65–99.72	3	1	0	0	1	1
Bacillus halotolerans	99.74	1	0	0	0	1	0
Bacillus licheniformis	99.79	1	0	1	0	0	0
Bacillus mobilis	99.86	1	1	0	0	0	0
Bacillus oryzaecorticis	99.72	1	0	1	0	0	0
Bacillus pumilus	99.79	1	0	0	1	0	0
Bacillus siamensis	98.80	1	1	0	0	0	0
Bacillus sp.	97.15–100	51	12	2	3	23	11
Bacillus subtilis	98.75–99.86	3	3	0	0	0	0
Bacillus thuringiensis	99.38–99.93	11	2	0	0	6	3
Bacillus velezensis	99.93	1	0	0	0	0	1
Bacillus wiedmannii	99.72	1	1	0	0	0	0
Bacterium strain	97.84–100	9	2	1	2	3	1
Brevibacillus choshinensis	99.86	1	0	0	0	0	1
Brevibacterium sp.	99.16–99.79	3	1	0	2	0	0
Paenibacillus sp.	99.24–99.65	2	1	0	0	0	1
Peribacillus frigoritolerans	96.98–99.79	9	4	0	2	3	0
Peribacillus huizhouensis	100	1	0	1	0	0	0
Peribacillus simplex	96.92–100	12	2	2	0	3	5
Peribacillus sp.	98.74–99.51	2	1	0	0	1	0
Priesta aryabhattai	98.28–100	5	4	0	0	0	1
Priesta megaterium	97.31–100	14	5	2	1	1	5
Psychrobacillus sp.	99.10	1	0	0	0	1	0
Solibacillus sp.	99.65	1	0	0	0	1	0
Uncultured bacterium	94.70–99.86	4	2	0	0	2	0

a The table gives an overview of representative isolates from the culturable olive sporobiota identified by 16S rRNA sequencing. The total number of isolates is indicated, as well as the number of isolates by origin. BG, Bedmar y Garcíez; JP, Jimena (Jaén); LGJ, La Guardia de Jaén; LIN, Linares (Jaén); MA, Málaga.

### Sporulation ability of identified isolates.

To screen for the ability to sporulate, bacterial isolates were plated on sporulation-inducing medium, and spore formation was observed after incubation at 30°C for 3 days. Microscopic examination demonstrated that all isolates were able to form endospores (data not shown). In most cases, mostly free spores were observed with a minority of cells still in the sporulation phase, i.e., phase bright spores in the mother cell or vegetative cells.

A small number of isolates showed a slower sporulation process with immature spores, i.e., unreleased, phase bright spores in the mother cell, as the predominant configuration. However, all isolates did demonstrate the ability to sporulate.

### Screening for B. anthracis virulence plasmids.

To screen for the presence of virulent B. anthracis among the olive orchard isolates, PCR analyses were performed to detect the virulence plasmids pXO1 and pXO2 (23). The results indicated the absence of virulent B. anthracis species for all isolates, although the presence of nonvirulent B. anthracis strains or genetically closely related Bacillus sp. strains could not be excluded (data not shown).

### Antibiotic susceptibility of identified isolates.

To test the susceptibility of the culturable olive sporobiota to antibiotic treatment, 117 isolates were selected based on their representative position in the Rep-PCR profiles and used in disk diffusion assays according to the European Committee on Antimicrobial Susceptibility Testing (EUCAST) method (2021) (Table 3; Fig. S1). Overall, the selected isolates were generally susceptible to gentamicin (GEN) (99.1%), nitrofurantoin (NFN) (96.6%), imipenem (IPM) (94.9%), tetracycline (TET) (88.0%), erythromycin (ERY) (86.3%), amoxicillin-clavulanic acid (AMC) (83.8%), ampicillin (AMP) (80.3%), and chloramphenicol (CHL) (79.5%). On the other hand, the isolates showed resistance to rifampicin (RA) (92.3%), while 3.4% of isolates were susceptible to this antibiotic, and 4.3% showed intermediate susceptibility.

**TABLE 3:** Antibiotic susceptibility of representative selected culturable olive sporobiota isolates^*a*^

Origin (No. tested isolates)	Susceptibility	TET	ERY	AMC	AMP	CHL	CIP	RA	NFN	GEN	IPM	CTX
LIN (58)	Susceptible	51	48	49	47	47	0	2	56	57	57	26
	Intermediate	1	0	5	2	0	56	1	1	0	0	3
	Resistant	6	10	4	9	11	2	55	1	1	1	29
JP (9)	Susceptible	9	8	9	9	6	0	0	9	9	9	1
	Intermediate	0	0	0	0	0	9	1	0	0	0	1
	Resistant	0	1	0	0	3	0	8	0	0	0	7
BG (9)	Susceptible	9	7	9	8	7	0	0	8	9	9	2
	Intermediate	0	0	0	1	0	7	1	1	0	0	0
	Resistant	0	2	0	0	2	2	8	0	0	0	7
LGJ (28)	Susceptible	24	28	21	20	23	0	2	27	28	25	4
	Intermediate	1	0	3	1	0	27	2	1	0	0	0
	Resistant	3	0	4	7	5	1	24	0	0	3	24
MA (13)	Susceptible	10	10	10	10	10	0	1	13	13	11	2
	Intermediate	0	0	1	0	0	13	0	0	0	0	0
	Resistant	3	3	2	3	3	0	12	0	0	2	11
TOTAL (117)	Susceptible	103	101	98	94	93	0	5	113	116	111	35
	Intermediate	2	0	9	4	0	112	5	3	0	0	4
	Resistant	12	16	10	19	24	5	107	1	1	6	78

a AMC, amoxicillin-clavulanic acid; AMP, ampicillin; CHL, chloramphenicol; CIP, ciprofloxacin; CTX, cefotaxime; ERY, erythromycin; GEN, gentamicin; IPM, imipenem; NFN, nitrofurantoin; RA, rifampicin; TET, tetracycline. BG, Bedmar y Garcíez; JP, Jimena (Jaén); LGJ, La Guardia de Jaén; LIN, Linares (Jaén); MA, Málaga.

Although to a lower extent than RA, the majority of tested isolates also showed resistance to cefotaxime (CTX) (66.7%), with 29.9% full and 3.4% intermediate susceptibility when exposed to this antibiotic. Interestingly, almost all isolates exhibited intermediate susceptibility to ciprofloxacin (CIP) (95.7%), while 4.3% were resistant to CIP treatment.

Conversely, a minority of the analyzed isolates were resistant to the antibiotics CHL (20.5%), AMP (16.2%), ERY (13.7%), TET (10.3%), AMC (8.5%), IPM (5.1%), NFN (0.9%), and GEN (0.9%), while an intermediate susceptibility was detected upon exposure to AMC (7.7%), AMP (3.4%), NFN (2.6%), and TET (1.7%). This overall trend was apparently independent of the sample origin.

### Tolerance of heavy metal exposure and mineral-based fertilizer treatment.

Finally, the tolerance to heavy metal exposure and mineral-based fertilizer treatment was tested, to evaluate resistance properties of the culturable olive sporobiota to these substances. For this, first, MICs for heavy metals were determined for 64 isolates chosen based on their representative position within the phylogenetic trees (Table 4). MIC was defined as the minimal concentration that inhibits visible bacterial growth (23). The results indicated that isolates of the olive sporobiota were able to tolerate relatively high heavy metal loads with concentrations ranging from 0.5 mM ≤ MIC < 25 mM. In this regard, results suggested the following order of metal tolerance for the tested isolates: iron > copper > nickel > manganese > zinc > cadmium. No particular strain patterns could be established, although in general B. cereus strains tended to display high resistance to iron and zinc, while P. megaterium appeared to mostly have high tolerance to manganese. P. simplex, on the other hand, consistently showed good tolerance toward zinc.

**TABLE 4:** Tolerance of selected representative olive culturable olive sporobiota isolates to heavy metals and mineral-based (NPK) olive fertilizer^*a*^

Origin	Isolate	Metal (MIC in mM)						NPK fertilizer		
		Iron	Manganese	Nickel	Copper	Zinc	Cadmium	0.1×	1×	10×
LIN	Bacillus amyloliquefaciens JXQZ11 (UJA_LIN_005)	25	25	10	10	10	0.5	+	++	+++
	Bacillus subtilis IARI-NIAW1-13 (UJA_LIN_009)	25	10	10	10	10	0.5	+	++	+++
	Peribacillus sp. strain QHPH60-1 (UJA_LIN_014)	25	10	10	10	10	0.5	+	+	−
	Brevibacterium frigoritolerans EH13 (UJA_LIN_020)	25	10	10	10	10	0.5	+	++	−
	Bacillaceae bacterium NR40 (UJA_LIN_027)	10	10	10	10	10	0.5	+	++	−
	Bacterium strain QLN201807IPB2 (UJA_LIN_029)	10	25	10	10	10	2	+	++	+
	Priesta aryabhattai BPR078 (UJA_LIN_030)	10	10	10	10	10	0.5	+	+	+
	Priesta megaterium NY-3 (UJA_LIN_037)	25	25	10	10	10	0.5	+	+	+
	Bacillus sp. strain CLC-M22 (UJA_LIN_043)	25	10	10	10	10	0.5	+	++	+
	Bacillus endophyticus Planc11 (UJA_LIN_056)	25	10	10	10	10	0.5	+	++	++
	Peribacillus simplex KLV34 (UJA_LIN_066)	10	10	10	10	25	10	+	++	+
	Bacillus mobilis KUBOTAB7 (UJA_LIN_077)	25	10	10	10	10	0.5	+	++	+
	Bacillus sp. strain SG13 (UJA_LIN_084)	10	10	10	10	25	10	+	++	+
	Bacillus sp. strain E7 (UJA_LIN_100)	25	10	10	10	10	0.5	+	+	+
	Priestia megaterium yangyueK8 (UJA_LIN_116)	10	10	10	10	25	10	+	++	+
	Priesta megaterium (UJA_LIN_117)	10	10	10	10	25	25	+	++	+
	Bacillus subtilis 262XY3 (UJA_LIN_129)	10	10	10	10	10	2	+	++	+++
	Bacillus aerius NRB050 (UJA_LIN_134)	10	10	10	10	10	0.5	+	+	−
	Bacillus amyloliquefaciens subsp. *plantarum* L11 (UJA_LINA_136)	25	10	10	10	2	0.5	+	++	+++
	Bacillus sp. strain Ht-q6 (UJA_LIN_145)	25	10	10	10	2	0.5	+	++	+++
JP	UJA_JP_148	25	10	10	10	25	2	+	+	−
	Bacillus oryzaecorticis ER14 (UJA_JP_150)	25	2	10	10	25	10	+	+	−
	Bacillus sp. strain LJ146 (UJA_JP_152)	25	2	2	10	25	2	+	++	+
	Peribacillus simplex QT-121 (UJA_JP_154)	10	10	10	10	25	2	+	+	+
	Bacillus licheniformis B-9 (UJA_JP_155)	25	10	25	25	25	10	+	+	−
	Bacillus sp. strain AB74 (UJA_JP_156)	10	10	25	10	25	10	+	++	−
	UJA_JP_157	10	25	25	10	10	10	+	+	−
	Priesta megaterium ZBHT36 (UJA_JP_159)	10	25	25	10	25	2	+	+	+
BG	Bacterium strain MTL5-77 (UJA_BG_169)	10	10	25	10	2	25	+	+	+
	Brevibacterium frigoritolerans PgBE249 (UJA_BG_171)	10	10	25	10	25	25	+	+	+++
	*Bacillus* sp. strain BDH4 (UJA_BG_175)	10	25	25	25	10	25	+	+	+
	Priesta megaterium SX6 (UJA_BG_176)	10	25	25	10	10	10	+	+	−
	Bacillus pumilus MCAS8 (UJA_BG_178)	10	25	25	10	25	10	+	+	+
	UJA_BG_182	10	25	25	10	2	10	+	+	+
	Brevibacterium sp. strain M-14 (UJA_BG_186)	10	10	25	10	10	10	+	++	+++
	Brevibacterium sp. strain 201705CJKOP-45 (UJA_BG_187)	10	10	25	10	2	10	+	+	−
LGJ	Bacillus sp. strain BH40 (UJA_LGJ_190)	25	10	10	25	10	2	+	+	++
	Bacillus sp. strain HBUM206332 (UJA_LGJ_194)	10	10	10	10	10	10	+	++	++
	Bacillus cereus ML101A (UJA_LGJ_199)	25	10	10	25	25	2	+	+	+
	Bacillus thuringiensis Gaoshi-1 (UJA_LGJ_204)	10	25	10	10	10	2	+	+	+
	Bacillus sp. strain ARB-SM12 (UJA_LGJ_210)	10	10	10	10	25	10	+	++	+
	Bacillus cereus MZ-1 (UJA_LGJ_213)	25	25	25	10	25	10	++	+	+
	Bacillus thuringiensis JC-3 (UJA_LGJ_216)	25	25	25	10	25	2	+	+	+
	Bacterium strain ANA_YJ_J19 (UJA_LGJ_223)	25	2	25	10	25	2	++	+	+
	Bacillus cereus ML101A (UJA_LGJ_229)	25	25	25	10	25	2	++	+	+
	Bacillus thuringiensis Gaoshi-1 (UJA_LGJ_233)	10	10	10	10	25	10	+	++	+
	Bacillus sp. strain Ha15 (UJA_LGJ_246)	25	25	25	10	25	2	+	+	+
	Bacillus cereus M2 (UJA_LGJ_258)	25	10	25	10	25	2	+	+	+
	Bacterium strain ANA_YJ_J34 (UJA_LGJ_320)	25	10	10	10	25	0.5	+	++	+++
	Bacillus cereus X1 (UJA_LGJ_323)	10	10	10	10	25	2	+	++	+++
	Bacillus thuringiensis BH49 (UJA_LGJ_337)	10	25	10	10	10	2	+	+	+
	Bacillus sp. strain B37 (2014) (UJA_LGJ_355)	25	10	10	10	25	0.5	+	+	−

MA	Bacillus sp. strain M112 (UJA_MA_360)	10	10	25	25	>10	>10	+	+	+
	Bacillus sp. strain DGL24 (UJA_MA_363)	10	25	25	10	>10	>10	+	+	+
	Peribacillus simplex EM-C3 (UJA_MA_373)	10	25	25	10	>10	>10	+	+	+
	UJA_MA_379	10	25	25	10	>10	>10	+	+	+
	Bacillus sp. strain CPO 4.230 (UJA_MA_382)	10	10	25	10	>10	>10	+	+	+
	Bacillus endophyticus YN14 (UJA_MA_395)	10	25	25	10	>10	>10	+	++	+
	Peribacillus simplex X10 (UJA_MA_398)	10	10	25	10	>10	>10	+	+	+
	Bacterium strain MTL5-77 (UJA_MA_403)	10	10	10	10	10	2	+	+	+
	Priesta megaterium B2 (UJA_MA_405)	25	25	10	25	10	10	+	+	+
	Bacillus thuringiensis L26 (UJA_MA_406)	25	10	10	25	25	2	+	+	+
	Bacillus velezensis K-13 (UJA_MA_417)	25	10	10	25	10	2	+	+	++

a NPK, nitrogen-phosphate-potassium; BG, Bedmar y Garcíez; JP, Jimena (Jaén); LGJ, La Guardia de Jaén; LIN, Linares (Jaén); MA, Málaga.

Second, the culturable olive sporobiota tolerance to mineral-based fertilizer treatment was evaluated. For this the same representative isolates as above were exposed to a nitrogen-phosphate-potassium (NPK) fertilizer, routinely used in olive agriculture. Bacterial growth after NPK exposure at different concentrations was evaluated in comparison to untreated controls (Table 4). The results showed that NPK treatment at 0.1× and 1× of the recommended concentration (0.3 and 3 g/liter, respectively) had no negative effect on bacterial growth. In fact, treatment with 1× NPK did improve the growth of 37.5% of Bacillus sp. (24 strains) in comparison to untreated samples. Exposure to higher-than-recommended concentrations of NPK fertilizer had a more varied effect. In this regard, 12 strains (18.8%) were susceptible to the treatment, while 4 strains (6.3%) and 9 strains (14.1%) showed increased or highly increased growth, respectively, compared to untreated samples. These data suggest that fertilizer treatment of olive orchards can also influence the growth properties of the bacterial community, which could have both positive and negative effects.

## DISCUSSION

The plant microbiome is essential to maintain a healthy soil and plant environment (2). Bacillus spp. and recently reclassified species previously belonging to the genus present one of the major bacterial components of agricultural ecosystems, including olive orchards (8, 24). As such, they play a central role in maintaining a healthy environment for the plant, for example by capturing nutrients and facilitating nutrient uptake, removing toxic elements, or acting as antimicrobial agents (8, 22). Given the importance of these bacteria in maintaining a healthy and productive plant environment, in this study, we aimed to determine and characterize the culturable spore-forming microbiome of Spanish olive orchards collectively called the culturable olive sporobiota.

The 16S rRNA analysis showed a wide variety of Bacillus sp. and Bacillus related species present in the soil surrounding olive trees, as well as the leave surface and endophytic community of olive trees. Bacillus spp. are ubiquitously present in the environment, including soil and plants (6). Dendrogram analysis showed a great diversity and heterogeneity of spore formers present in olive agricultural soils and olive plants. Rep-PCR fingerprinting of the culturable sporobiota isolates from different origins revealed various genetic groups of each origin (between 2 and 4). It is, however, noteworthy that a variety of species were detected throughout the sample collection, irrespective of the origin. Consequently, the overall presence and distribution of *Bacilli* and the great diversity of different species were consistent with previous reports on microbial communities for agricultural soil samples (25). Although in general an even distribution of detected sporobiota isolates was observed in the Rep-PCR profiles, isolates from the LGJ origin (La Guardia de Jaén) showed a higher prevalence of members of the B. cereus sensu lato group (excluding virulent B. anthracis) with 15 of 24 B. cereus sensu lato isolates originating from this orchard. Like its fellow Bacillus species, B. cereus sensu lato is ubiquitously present in the environment, including soil (26), while the prevalence in this origin could be due to soil conditions particularly conducive to B. cereus sensu lato. In this regard, B. cereus sensu lato was also the most identified species group (24 isolates) in the culturable olive sporobiota (B. cereus sp. [12 isolates], B. thuringiensis [11 isolates], and B. wiedmannii [1 isolate]). Multiplex PCR analysis for virulent pXO1 and pXO2 plasmids furthermore indicated the absence of virulent B. anthracis strains among the culturable olive sporobiota (27). However, the presence of nonvirulent strains in our culturable olive sporobiota collection cannot be confidently excluded under the chosen experimental conditions (28).

In addition, we could observe slight variability in bacterial load depending on the origin. This is probably not surprising, as bacterial load depends on the soil composition and also experiences seasonal fluctuations (29).

The inclusion of a heat treatment step (78°C, 20 min) during bacterial isolation ensured that only heat-resistant, presumably spore-forming bacteria were isolated (30). The formation of endospores, conferring the ability to resist harsh environmental such as wet heat (31), was confirmed by the ability of all tested isolates to sporulate on rich sporulation media. Implicitly, this procedure also shows that all isolated strains were able to successfully undergo sporulation/germination life cycles, as in this work both dormant and vegetative cells were studied (32).

Antibiotic resistance among microbial communities is a growing global concern for agriculture (33) with major European Union and international policy initiatives aiming to limit its spread (34). Hence, in this study, we also evaluated the resistance of the culturable olive sporobiota to selected antibiotics. Our results confirmed those obtained in other studies, with tested Bacillus spp. isolates being susceptible to gentamicin (99.1%), nitrofurantoin (96.6%), imipenem (94.9%), tetracycline (88.0%), erythromycin (86.3%), and chloramphenicol (CHL) (79.5%) (35). In contrast to other reports, the tested isolates also showed relatively high susceptibility to several β-lactam antibiotics, i.e., amoxicillin-clavulanic acid (83.8%) and ampicillin (80.3%), while the expected the resistance to cefotaxime (66.7%)—although to a lower extent—was detected (36, 37). It would thus be interesting to test the presence/absence of β-lactamase enzymes in the bacterial genome in particular of these isolates in future studies.

In addition, and in accordance with previous studies, the tested isolates showed high resistance to rifampicin (92,3%) (36). Interestingly, and in contrast to other studies, mostly intermediate resistance to ciprofloxacin (95,7%) was detected for the tested isolates. This could potentially suggest that the members of the culturable olive sporobiota are acquiring a resistance to this antibiotic (35). It is also noteworthy that the overall trend of antibiotic resistance was also reflected when analyzed by origin. Hence, under the chosen conditions, the sporobiota origin did not influence antibiotic resistance properties of the tested representative isolates in our study.

In addition to AMR spread, heavy metal contamination is a serious challenge for commercial agriculture and food production, as the metals can be taken up by plants and ultimately enter the food chain (38). The European Union is thus constantly revising the safety of metal content in foodstuff and (if necessary) adjusting maximum permitted levels as set by Commission Regulation 1881/2006. The ability of Bacillus spp. to tolerate metal exposure, as well as absorb and remove heavy metals from the soil, thus effectively decontaminating the environment, has previously been shown (39). Hence, here we tested the olive sporobiota’s tolerance to heavy metal exposure. In accordance with literature on metal resistance of Bacillus and Lactobacillus spp., isolates showed a good tolerance to heavy metals, with an order of tolerance of iron > copper > nickel > manganese > zinc > cadmium (23). This suggests that members of the culturable olive sporobiota could also potentially thrive in soils with an elevated metal load as a result of environmental conditions or anthropogenic activity (40). Accordingly, as a next step, it would be interesting to test the metal remediation capacity of the most promising species isolated in this work.

Finally, we investigated the culturable olive sporobiota’s response to mineral-based fertilizer treatment, notably at low (0.1×), recommended (1×), and excess (10×) concentrations. Although there is a shift toward organic farming also in olive agriculture, still many of the most commonly used fertilizers are mineral based (https://ec.europa.eu/eurostat/statistics-explained/index.php?title=Agri-environmental_indicator_-_mineral_fertiliser_consumption, June 2022). In general, it has been shown that in addition to improving crop yields, mineral fertilizer application alters soil properties and the soil microbiome (41). In this regard, the applied fertilizers not only have beneficial effects on plant growth but also provide an extra source of nutrients to the bacterial community, consequently increasing microbial biomass and diversity (42). Findings from our study are in line with these expectations, showing an improved bacterial growth of over one third of tested olive sporobiota isolates (37.5%) and no negative effect in comparison to untreated samples at 1× concentration. These results indicate that mineral fertilizer treatment has a nonuniform moderately positive effect on the culturable sporobiota bacterial community in olive orchards when applied at the recommended concentration. Nevertheless, treatment at higher NPK concentrations (10×) had a negative effect on nearly one fifth (18.8%) of the tested isolates, suggesting a reduced tolerance of the culturable olive sporobiota to high NPK concentrations.

### Conclusions.

In this study, we determined the culturable Bacillus spp. and related spore-forming species in Spanish olive orchards collectively called the culturable olive sporobiota. We could demonstrate that the culturable olive sporobiota mostly follows susceptibility patterns for antibiotic treatments and heavy metal tolerance, although some strains differed from the expected pattern. We also showed that treatment with mineral fertilizer does not negatively affect or can even be conducive for bacterial growth. The obtained information may prove useful for further evaluating the effect and progression of AMR spread among microbial communities, as well as heavy metal tolerance in (contaminated) soils. Here, it would be particularly interesting for future investigations to study the ability of the culturable olive sporobiota collectively or separately to absorb and remove heavy metals from the soil or the potential effect of different farming practices (organics versus nonorganic) on the Bacillus spp. and related bacterial community.

It is also conceivable that one or several of the determined inherent species could play an important role in the development of novel biotechnological applications for agricultural use. In particular, the generated library of spore formers native to olive groves could provide a unique tool for the development of novel biological plant protection products in olive agriculture, notably given that Bacillus spp. already play a central role as one of the most widely used biopesticides, as well as its ability to produce a wide range of antimicrobial peptides (17, 21).

## MATERIALS AND METHODS

### Sampling in olive groves.

The samples analyzed in this study were collected over a period of 5 months (September 2021 to January 2022) from olive groves at five different locations in Andalusia, Spain (Table 1). Briefly, soil samples were taken close to the tree trunk at approximately 10-cm depths. Equally, leaf samples corresponding to the same vertical location on the tree were taken in the middle part of the canopy. The samples were collected in sterile containers and stored at room temperature until further processing.

### Isolation of heat-resistant bacteria.

To remove heat-labile, non-spore-forming organisms, 1 g of soil or 2 g of leaves were resuspended in 9 mL sterile 0.85% (wt/vol) saline solution and incubated at 78°C for 20 min. Serial dilutions were prepared in 0.85% (wt/vol) saline solution and plated in triplicate on tryptic soy agar (TSA) and incubated at 30°C overnight. Representative single colonies were randomly picked and purified on nonselective medium prior to storage in 25% (vol/vol) glycerol at −20°C or −80°C for further use. To isolate endophytic *Bacilli*, 2 g of leaves were first sterilized by serial washing steps in sterile 0.85% saline solution, 70% (vol/vol) ethanol, and 0.85% (wt/vol) saline solution prior to resuspension in 9 mL sterile 0.85% (wt/vol) saline solution. Endophytic bacteria were physically released by treatment in a stomacher unit (1 to 2 min, setting: high). Heat-resistant microorganisms were isolated from the resulting suspensions as described above.

### Molecular characterization and identification of the culturable olive sporobiota.

**(i) Detection of virulent Bacillus anthracis species.** In a first instance, PCR was used to screen for the potential presence of virulent B. anthracis species and take necessary safety precautions, if applicable. To do so, a multiplex PCR as described by Ogawa et al. (27) was performed detecting *cap* and *pag* genes on the B. anthracis virulence plasmids pX01 and pX02, as well as targeting the chromosome of B. anthracis and B. anthracis-like species. PCR was performed from a fresh overnight colony using MyTaq Red Mix (Meridian Bioscience) following the manufacturer’s instructions under the following conditions: initial denaturation at 94°C for 1 min followed by 35 cycles of denaturation at 94°C for 30 s, annealing at 58°C for 2 min, extension at 72°C for 2 min, and final extension at 72°C for 10 min. Multiplex primers and concentrations are indicated in Table 5. PCR products were visualized by agarose gel electrophoresis (1% [wt/vol] agarose in 1× Tris-borate-EDTA buffer, 90 V, 45 min), stained with RedSafe nucleic acid staining solution (iNtRON), and visualized using a UV transilluminator.

**TABLE 5:** Primers used for detecting virulent B. anthracis species^*a*^

Primer	Sequence (5′ to 3′)	Concn	Target gene	Product size	Reference
BA_5031-F6	CGATGTAAATTCGGCACTGGATCTTC	0.6 μM	BA_5031	1,027 bp	Bacillus anthracis strain Ames
BA_5031-R6	TTCTATCATTCTTAGTGGAATGTGG				
16S-663F	AKGTGTAGCGGTGAAATGCGTAG	0.05 μM	16S rRNA	733 bp	Bacillus anthracis strain Ames 16S ribosomal RNA gene
16S-1395R	TGGTGTGACGGGCGGTGTGTACAAGG				
CAP-F8	TCATCCGGATCCAGGAGCAATGAG	0.2 μM	*cap*	578 bp	Bacillus anthracis strain TE702 encapsulation protein gene plasmid pX02
CAP-R7	GCAGGTAAAATACCTGTTCTTTCTG				
PA-F6	CCTTGTGGCAGCTTATCCGA	0.05 μM	*pag*	364 bp	Bacillus anthracis strain Sterne plasmid pX01
PA-R6	GTAGATTGGAGCCGTCCCAG				
ACTB-F3813	CAGATCATGTTYGAGACCTTCAACAC	0.2 μM	ACTB	224 bp	Homo sapiens *actin*, *beta* gene
ACTB-R3813	TCVGTSAGGATCTTCATGAGGTAGTC				
hBC/BT-F	ATTTCTTTACTCATAGATAAATCACCA	0.1 μM	BACI_c47770	197 bp	Bacillus cereus var. anthracis strain Cl
hBC/BT-R	GAAATYCCATCAGTTGCAAGTGAGTTG				

a The table lists the primers used for detecting the presence of the virulent B. anthracis species in the culturable olive sporobiota isolated in this study (adapted from Ogawa et al. [27]).

**(ii) Rep-PCR fingerprinting.** A total of 407 candidates were characterized by Rep-PCR as described by Abriouel et al. (43). Essentially, DNA was amplified from a fresh overnight single colony using 1 μL of 100 μM (GTG)_5_ primer (5′-GTGGTGGTGGTGGTG-3′) and MyFi polymerase (meridian Bioscience) in a final volume of 25 μL using the following conditions: initial denaturation 95°C for 3 min, followed by 30 cycles of denaturation at 90°C for 30 s, annealing step 1 at 40°C for 1 min, annealing step 2 at 40°C to 72°C for 5:20 min (ramping 0.1°C/s) and extension at 72°C for 2 min. A final extension was performed at 72°C for 8 min. Amplicons were subsequently subjected to agarose gel electrophoresis (1.8% [wt/vol] agarose in 1× Tris-borate-EDTA buffer) for 16 h at 48 V, stained with RedSafe nucleic acid staining solution (iNtRON) and visualized using a UV transilluminator. Band profiles were analyzed using Bionumerics software, version 2.5 (Applied Maths, Kortrijk, Belgium). The Pearson product correlation coefficient and the unweighted pair group method using arithmetic averages (UPGMA) were used to group Rep-PCR patterns through cluster analysis.

**(iii) Identification of the culturable olive sporobiota.** Representative isolates of each profile as identified by Rep-PCR and morphological features were selected and identified by 16S rRNA gene sequencing. Bacterial cultures were grown at 37°C overnight in tryptic soy broth, washed with 0.5% (wt/vol) saline solution, boiled for 10 min, and immediately transferred on ice. The resulting genomic DNA extracts were centrifuged, and 1 μL supernatant was used for PCR amplification with the primer pair 27f-YM/1492r (5′-AGAGTTTGATYMTGGCTCAG-3′/5′-TACCTTGTTACGACTT-3′) (44).

Purified amplicons were sequenced using the same primer pair, and partial sequences were assembled with the A plasmid editor (ApE) software (45). Bacterial species were tentatively identified through a homology search with the BLAST N algorithm (National Center for Biotechnology Information [NCBI], USA) based on the highest alignment score and the percentage of identity.

### Evaluation of sporulation ability.

To confirm the ability to sporulate under laboratory conditions, culturable sporobiota isolates were subjected to nutrient starvation to induce sporulation (46). Briefly, a fresh colony was streaked on 2× SG plates and incubated at 30°C for 3 days. The ability of the isolate to form endospores was evaluated by light microscopy using a 100× objective in a Zeiss upright light microscope. Sporulation efficiency was evaluated based on visual release of endospores from mother cells.

### Antibiotic susceptibility testing.

Antibiotic susceptibility of the isolated olive sporobiota species was examined by the disk diffusion method as described by the EUCAST 2021 guidelines. Essentially, bacterial suspensions were plated to form confluent lawns on Mueller-Hinton agar, and antibiotic disks were placed on the plates. The tested antibiotics were ampicillin (AMP, 10 μg), amoxicillin-clavulanic acid (AMC, 20/10 μg), cefotaxime (CTX, 30 μg), chloramphenicol (CHL, 30 μg), ciprofloxacin (CIP, 5 μg), rifampicin (RA, 5 μg), nitrofurantoin (NFN, 300 μg), erythromycin (ERY, 15 μg), gentamicin (GEN, 10 μg), imipenem (IPM, 10 μg), and tetracycline (TET, 30 μg), respectively. After 18 to 20 h incubation at 35°C, the inhibition zones were measured and compared to the breaking points of the respective antibiotics. For IPM, CIP, and ERY, Bacillus specific breakpoint values set by EUCAST (2021) were used. As there were no Bacillus-specific values available for the remaining antibiotics, the EUCAST criteria (2021) for Staphylococcus aureus were adopted for TET, GEN, RA, and CHL, whereas for AMC, AMP, NFN, and CTX, the *Enterobacteriales* breakpoints as defined by the Clinical and Laboratory Standards Institute (CLSI, 2020) were used as references (35).

### Evaluation of resistance properties.

**(i) Metal tolerance.** The sensitivity of selected sporobiota isolates, representative of identified strains/origins toward the heavy metals cadmium (CdSO_4_·8/3H_2_O), copper (CuCl_2_·2H_2_O), iron (FeSO_4_·7H_2_O), zinc (ZnCl_2_), nickel (NiCl_2_·2H_2_O), and manganese (MnCl_2_·4H_2_O) (all Sigma-Aldrich, ES) was tested using the broth microdilution method (23). A total of 180 μL Mueller-Hinton broth supplemented with the respective metal at concentrations ranging from 0 to 25 mM were distributed in each well of 96-well microtiter plates. Metal solutions were then inoculated with 20-μL bacterial overnight cultures grown in tryptic soy broth or agar at 37°C previously adjusted to a concentration of 0.5 McFarland in Mueller-Hinton Broth. Microtiter plates were incubated at 37°C under aerobic conditions, and bacterial growth was evaluated by the presence of turbidity and/or bacterial deposition at the bottom of the plate. MIC was defined as the lowest concentration of metal that inhibited visible growth. Each experiment was performed in triplicate.

**(ii) Fertilizer tolerance.** Tolerance to a commercial nitrogen-phosphorus-potassium-based mineral fertilizer (NPK 20-8-14) (Abono Olivos, Massó Garden) was evaluated. The fertilizer was prepared following the manufacturer’s instructions in Mueller-Hinton broth at concentrations of 0.3 g/liter (0.1×), 3 g/liter (1×), and 30 g/liter (10×). The susceptibility of fertilizer treatment of selected olive sporobiota isolates was evaluated as described above.
