# Supplementary material for: Characterization of the Culturable Sporobiota of Spanish Olive Groves and Its Tolerance toward Environmental Challenges
Source: Microbiol Spectr. 2023 Jan 31;11(2):e04013-22. doi: 10.1128/spectrum.04013-22 (PMC10100736; doi:10.1128/spectrum.04013-22)

Supplemental table 1: List of selected isolates of the culturable olive sporobiota by origin identified by 16S rRNA sequencing.

Origin	Sample ID	Identified *Bacillus* species (Blast-ID)	% Identity
**LIN**	**UJA_LIN_004**	*Priestia aryabhattai* strain H7BC2 16S ribosomal RNA gene, partial sequence	100
	**UJA_LIN_005**	*Bacillus amyloliquefaciens* strain JXQZ11 16S ribosomal RNA gene, partial sequence	99.79
	**UJA_LIN_007**	*Priestia megaterium* strain TIRB11 16S ribosomal RNA gene, partial sequence	100
	**UJA_LIN_009**	*Bacillus subtilis* strain IARI-NIAW1-13 16S ribosomal RNA gene, partial sequence	98.75
	**UJA_LIN_014**	*Peribacillus* sp. strain QHPH60-1 16S ribosomal RNA gene, partial sequence	99.51
	**UJA_LIN_017**	Bacterium strain BS1518 16S ribosomal RNA gene, partial sequence	99.65
	**UJA_LIN_019**	*Bacillus* sp. SPT-W8 16S ribosomal RNA gene, partial sequence	99.79
	**UJA_LIN_020**	*Brevibacterium frigoritolerans* strain EH13 16S ribosomal RNA gene, partial sequence	99.72
	**UJA_LIN_023**	*Brevibacterium frigoritolerans* strain QT-70 16S ribosomal RNA gene, partial sequence	98.04
	**UJA_LIN_026**	*Bacillus* sp. strain 201705CJKOP-7 16S ribosomal RNA gene, partial sequence	99.86
	**UJA_LIN_027**	*Bacillaceae* bacterium NR40 16S ribosomal RNA gene, partial sequence	88,48
	**UJA_LIN_029**	Bacterium strain QLN201807IPB2 16S ribosomal RNA gene, partial sequence	99.58
	**UJA_LIN_030**	*Priestia aryabhattai* strain BPR078 16S ribosomal RNA gene, partial sequence	99.72
	**UJA_LIN_031**	*Priestia aryabhattai* strain Hc15 16S ribosomal RNA gene, partial sequence	100
	**UJA_LIN_032**	*Bacillus wiedmannii* strain GB101 16S ribosomal RNA gene, partial sequence	99.72
	**UJA_LIN_035**	*Bacillus cereus* strain Se05 16S ribosomal RNA gene, partial sequence	98.16
	**UJA_LIN_037**	*Priesta megaterium* strain NY-3 16S ribosomal RNA gene, partial sequence	99.72
	**UJA_LIN_043**	*Bacillus* sp. (in: Bacteria) strain CLC-M22 16S ribosomal RNA gene, partial sequence	100
	**UJA_LIN_047**	*Bacillus atrophaeus* strain F3-60 16S ribosomal RNA gene, partial sequence	97.76
	**UJA_LIN_049**	*Peribacillus simplex* strain ER20 16S ribosomal RNA gene, partial sequence	99.93
	**UJA_LIN_052**	*Priesta megaterium* strain ML257 16S ribosomal RNA gene, partial sequence	99.86
	**UJA_LIN_054**	*Bacillus* sp. strain 201705CJKOP-20 16S ribosomal RNA gene, partial sequence	99.72
	**UJA_LIN_055**	*Peribacillus frigoritolerans* strain QT343 16S ribosomal RNA gene, partial sequence	99.58
	**UJA_LIN_056**	*Bacillus endophyticus* strain Planc11 16S ribosomal RNA gene, partial sequence	99.72

	**UJA_LIN_063**	*Brevibacterium* sp. strain CJKOP-132 16S ribosomal RNA gene, partial sequence	99.79
	**UJA_LIN_064**	*Bacillus* sp. strain SRD03 16S ribosomal RNA gene, partial sequence	100
	**UJA_LIN_065**	Uncultured bacterium isolate 1112864242278 16S ribosomal RNA gene, partial sequence	94.70
	**UJA_LIN_066**	*Peribacillus simplex* strain KLV34 16S ribosomal RNA gene, partial sequence	99.86
	**UJA_LIN_067**	*Bacillus* sp. strain YB-3 16S ribosomal RNA gene, partial sequence	99.59
	**UJA_LIN_073**	*Bacillus thuringiensis* strain EGI78 16S ribosomal RNA gene, partial sequence	99.65
	**UJA_LIN_076**	*Bacillus cereus* strain MLY1 chromosome MLY1.0, complete sequence	99.31
	**UJA_LIN_077**	*Bacillus mobilis* strain KUBOTAB7 16S ribosomal RNA gene, partial sequence	99.86
	**UJA_LIN_080**	*Bacillus thuringiensis* strain BH49 16S ribosomal RNA gene, partial sequence	99.86
	**UJA_LIN_083**	*Bacillus* sp. (in: Bacteria) strain Q16OP1 16S ribosomal RNA gene, partial sequence	100
	**UJA_LIN_084**	*Bacillus* sp. SG13 16S ribosomal RNA gene, partial sequence	97.84
	**UJA_LIN_085**	*Bacillus* sp. strain Z88 16S ribosomal RNA gene, partial sequence	100
	**UJA_LIN_087**	*Bacillus cereus* strain B19 16S ribosomal RNA gene, partial sequence	99.79
	**UJA_LIN_089**	*Priestia aryabhattai* strain S22 16S ribosomal RNA gene, partial sequence	98.28
	**UJA_LIN_098**	*Bacillus* sp. strain XA15-12 16S ribosomal RNA gene, partial sequence	99.72
	**UJA_LIN_100**	*Bacillus* sp. E7 16S ribosomal RNA gene, partial sequence	99.93
	**UJA_LIN_106**	Uncultured bacterium gene for 16S rRNA, partial sequence, clone: G2CLN31	99.86
	**UJA_LIN_107**	*Peribacillus frigoritolerans* strain FJAT-44608 16S ribosomal RNA gene, partial sequence	96.98
	**UJA_LIN_110**	*Paenibacillus* sp. X6 16S ribosomal RNA gene, partial sequence	99.24
	**UJA_LIN_116**	*Priestia megaterium* strain yangyueK8 16S ribosomal RNA gene, partial sequence	99.86
	**UJA_LIN_117**	*Priestia megaterium* 16S ribosomal RNA gene, partial sequence	97.31
	**UJA_LIN_124**	*Bacillus subtilis* strain WR10-qm 16S ribosomal RNA gene, partial sequence	99.86
	**UJA_LIN_129**	*Bacillus subtilis* strain 262XY3 16S ribosomal RNA gene, partial sequence	99.86
	**UJA_LIN_131**	*Bacillus altitudinis* strain SH124 16S ribosomal RNA gene, partial sequence	99.86
	**UJA_LIN_134**	*Bacillus aerius* strain NRB050 16S ribosomal RNA gene, partial sequence	98.20

	**UJA_LIN_136**	*Bacillus amyloliquefaciens* subsp. plantarum strain L11 16S ribosomal RNA gene, partial sequence	99.93
	**UJA_LIN_139**	*Bacillus siamensis* strain CL-2 16S ribosomal RNA gene, partial sequence	98.80
	**UJA_LIN_145**	*Bacillus* sp. Ht-q6 16S ribosomal RNA gene, partial sequence	99.65
**JP**	**UJA_JP_147**	*Peribacillus huizhouensis* strain QT434 16S ribosomal RNA gene, partial sequence	100
	**UJA_JP_150**	*Bacillus oryzaecorticis* strain ER14 16S ribosomal RNA gene, partial sequence	99.72
	**UJA_JP_151**	*Peribacillus simplex* strain KLT9 16S ribosomal RNA gene, partial sequence	99.86
	**UJA_JP_152**	*Bacillus* sp. strain LJ146 16S ribosomal RNA gene, partial sequence	99.58
	**UJA_JP_153**	Bacterium strain BS0460 16S ribosomal RNA gene, partial sequence	99.72
	**UJA_JP_154**	*Peribacillus simplex* strain QT-121 16S ribosomal RNA gene, partial sequence	99.86
	**UJA_JP_155**	*Bacillus licheniformis* strain B-9 16S ribosomal RNA gene, partial sequence	99.79
	**UJA_JP_156**	*Bacillus* sp. AB74 16S ribosomal RNA gene, partial sequence	99.93
	**UJA_JP_159**	*Priestia megaterium* strain ZBHT36 16S ribosomal RNA gene, partial sequence	99.17
	**UJA_JP_162**	*Priestia megaterium* strain CS33 16S ribosomal RNA gene, partial sequence	95.18
**BG**	**UJA_BG_165**	*Bacillus* sp. (in: Bacteria) strain Chl-1a 16S ribosomal RNA gene, partial sequence	99.65
	**UJA_BG_168**	*Bacillus* sp. WYT002 16S ribosomal RNA gene, partial sequence	99.93
	**UJA_BG_169**	Bacterium strain MTL5-77 16S ribosomal RNA gene, partial sequence	99.51
	**UJA_BG_171**	*Peribacillus frigoritolerans* strain PgBE249 16S ribosomal RNA gene, partial sequence	99.65
	**UJA_BG_172**	Bacterium strain BS0446 16S ribosomal RNA gene, partial sequence	100
	**UJA_BG_175**	*Bacillus* sp. BDH4 16S ribosomal RNA gene, partial sequence	99.86
	**UJA_BG_176**	*Priestia megaterium* strain SX6 16S ribosomal RNA gene, partial sequence	99.58
	**UJA_BG_178**	*Bacillus pumilus* strain MCAS8 16S ribosomal RNA gene, partial sequence	99.79
	**UJA_BG_183**	*Peribacillus frigoritolerans* strain TSS139 16S ribosomal RNA gene, partial sequence	99.79
	**UJA_BG_186**	*Brevibacterium* sp. strain M-14 16S ribosomal RNA gene, partial sequence	99.37
	**UJA_BG_187**	*Brevibacterium* sp. strain 201705CJKOP-45 16S ribosomal RNA gene, partial sequence	99.16
**LGJ**	**UJA_LGJ_190**	*Bacillus* sp. strain BH40 16S ribosomal RNA gene, partial sequence	99.58

	**UJA_LGJ_194**	*Bacillus* sp. (in: Bacteria) strain HBUM206332 16S ribosomal RNA gene, partial sequence	99.45
	**UJA_LGJ_199**	*Bacillus cereus* strain ML101A 16S ribosomal RNA gene, partial sequence	99.23
	**UJA_LGJ_201**	Uncultured bacterium clone N5 16S ribosomal RNA gene, partial sequence	99.79
	**UJA_LGJ_202**	*Peribacillus frigoritolerans* strain TS30 16S ribosomal RNA gene, partial sequence	99.65
	**UJA_LGJ_204**	*Bacillus thuringiensis* strain Gaoshi-1 16S ribosomal RNA gene, partial sequence	99.72
	**UJA_LGJ_206**	*Peribacillus simplex NBRC 15720 = DSM 1321 chromosome, complete genome*	99.65
	**UJA_LGJ_210**	*Bacillus* sp. strain ARB-SM12 16S ribosomal RNA gene, partial sequence	99.58
	**UJA_LGJ_213**	*Bacillus cereus* strain MZ-1 16S ribosomal RNA gene, partial sequence	99.72
	**UJA_LGJ_214**	*Bacillus* sp. strain Z105 16S ribosomal RNA gene, partial sequence	99.93
	**UJA_LGJ_216**	*Bacillus thuringiensis* strain JC-3 16S ribosomal RNA gene, partial sequence	99.93
	**UJA_LGJ_221**	*Bacillus* sp. B37(2014) 16S ribosomal RNA gene, partial sequence	99.79
	**UJA_LGJ_223**	Bacterium strain ANA_YJ_J19 16S ribosomal RNA gene, partial sequence	100
	**UJA_LGJ_224**	*Bacillus* sp. (in: Bacteria) strain DN7 16S ribosomal RNA gene, partial sequence	99.59
	**UJA_LGJ_225**	*Bacillus* sp. strain Z105 16S ribosomal RNA gene, partial sequence	99.58
	**UJA_LGJ_227**	*Bacillus* sp. SPT-W8 16S ribosomal RNA gene, partial sequence	99.86
	**UJA_LGJ_229**	*Bacillus cereus* strain ML101A 16S ribosomal RNA gene, partial sequence	99.79
	**UJA_LGJ_231**	*Bacillus endophyticus* strain IHB B 10265 16S ribosomal RNA gene, partial sequence	99.72
	**UJA_LGJ_233**	*Bacillus thuringiensis* strain Gaoshi-1 16S ribosomal RNA gene, partial sequence	99.79
	**UJA_LGJ_234**	*Bacillus thuringiensis* strain EGI78 16S ribosomal RNA gene, partial sequence	99.38
	**UJA_LGJ_235**	*Bacillus thuringiensis* strain ex4 16S ribosomal RNA gene, partial sequence	99.45
	**UJA_LGJ_237**	*Bacillus* sp. S20609 16S ribosomal RNA gene, partial sequence	99.65
	**UJA_LGJ_239**	*Peribacillus simplex* strain WJB70 16S ribosomal RNA gene, partial sequence	99.44
	**UJA_LGJ_240**	*Bacillus* sp. strain XRK21 16S ribosomal RNA gene, partial sequence	99.52
	**UJA_LGJ_246**	*Bacillus* sp. Ha15 16S ribosomal RNA gene, partial sequence	99.86
	**UJA_LGJ_248**	*Bacillus* sp. S12810 16S ribosomal RNA gene, partial sequence	99.45
	**UJA_LGJ_249**	*Peribacillus frigoritolerans* strain PgBE249 16S ribosomal RNA gene, partial sequence	99.65

	**UJA_LGJ_254**	*Bacillus* sp. (in: Bacteria) strain IC-1C2 16S ribosomal RNA gene, partial sequence	99.72
	**UJA_LGJ_257**	*Peribacillus simplex* strain S122 16S ribosomal RNA gene, partial sequence	100
	**UJA_LGJ_258**	*Bacillus cereus* strain M2 16S ribosomal RNA gene, partial sequence	99.45
	**UJA_LGJ_263**	*Peribacillus frigoritolerans* strain KM15 16S ribosomal RNA gene, partial sequence	99.58
	**UJA_LGJ_265**	*Bacillus* sp. strain M72 16S ribosomal RNA gene, partial sequence	99.79
	**UJA_LGJ_266**	*Bacillus* sp. B37(2014) 16S ribosomal RNA gene, partial sequence	97.15
	**UJA_LGJ_272**	*Peribacillus* sp. strain Ba-10 16S ribosomal RNA gene, partial sequence	98.74
	**UJA_LGJ_276**	*Bacillus cereus* strain JMG-03 16S ribosomal RNA gene, partial sequence	99.79
	**UJA_LGJ_277**	*Bacillus halotolerans* strain SY1836 16S ribosomal RNA gene, partial sequence	99.74
	**UJA_LGJ_280**	*Bacillus sp. (in: Bacteria) strain CPO 4.230 16S ribosomal RNA gene, partial sequence*	99.79
	**UJA_LGJ_282**	*Bacillus cereus* strain QH1 16S ribosomal RNA gene, partial sequence	99.93
	**UJA_LGJ_288**	*Bacillus* sp. (in: Bacteria) strain CPO 4.230 16S ribosomal RNA gene, partial sequence	99.65
	**UJA_LGJ_295**	*Bacillus* sp. enrichment culture clone SYW23 16S ribosomal RNA gene, partial sequence	99.52
	**UJA_LGJ_315**	*Bacillus* sp. 210_24 16S ribosomal RNA gene, partial sequence	99.79
	**UJA_LGJ_320**	Bacterium strain ANA_YJ_J34 16S ribosomal RNA gene, partial sequence	100
	**UJA_LGJ_323**	*Bacillus cereus* strain X1 16S ribosomal RNA gene, partial sequence	95.28
	**UJA_LGJ_324**	Bacterium YTN085712 from China 16S ribosomal RNA gene, partial sequence	98.07
	**UJA_LGJ_331**	*Psychrobacillus* sp. strain 206302 16S ribosomal RNA gene, partial sequence	99.10
	**UJA_LGJ_333**	*Bacillus* sp. (in: Bacteria) strain ATCC 13368 16S ribosomal RNA gene, partial sequence	99.24
	**UJA_LGJ_334**	*Bacillus* sp. strain M72 16S ribosomal RNA gene, partial sequence	99.93
	**UJA_LGJ_337**	*Bacillus thuringiensis* strain BH49 16S ribosomal RNA gene, partial sequence	99.65
	**UJA_LGJ_340**	*Bacillus cereus* strain ZF9 16S ribosomal RNA gene, partial sequence	99.51
	**UJA_LGJ_341**	*Bacillus cereus* strain 151gite 16S ribosomal RNA gene, partial sequence	99.86
	**UJA_LGJ_342**	Uncultured bacterium clone QHL10 16S ribosomal RNA gene, partial sequence	99.58
	**UJA_LGJ_349**	*Priestia megaterium* strain PSC1 16S ribosomal RNA gene, partial sequence	99.58

	**UJA_LGJ_352**	*Bacillus* sp. (in: Bacteria) strain C12 16S ribosomal RNA gene, partial sequence	99.79
	**UJA_LGJ_355**	*Bacillus* sp. B37(2014) 16S ribosomal RNA gene, partial sequence	99.93
	**UJA_LGJ_358**	*Solibacillus* sp. strain C41 16S ribosomal RNA gene, partial sequence	99.65
**MA**	**UJA_MA_360**	*Bacillus* sp. (in: Bacteria) strain M112 16S ribosomal RNA gene, partial sequence	99.86
	**UJA_MA_361**	*Bacillus sp*. enrichment culture clone SYW29 16S ribosomal RNA gene, partial sequence	99.86
	**UJA_MA_362**	*Bacillus* sp. LTY060901 16S ribosomal RNA gene, partial sequence	99.79
	**UJA_MA_363**	*Bacillus* sp. (in: Bacteria) strain DGL24 16S ribosomal RNA gene, partial sequence	98.97
	**UJA_MA_364**	*Priestia megaterium* strain SX6 16S ribosomal RNA gene, partial sequence	100
	**UJA_MA_366**	*Peribacillus simplex* strain EM-C3 16S ribosomal RNA gene, partial sequence	99.44
	**UJA_MA_369**	*Bacillus thuringiensis* strain 263AG8 16S ribosomal RNA gene, partial sequence	99.72
	**UJA_MA_371**	*Bacillus thuringiensis* strain 263AG8 16S ribosomal RNA gene, partial sequence	99.72
	**UJA_MA_373**	*Peribacillus simplex* strain EM-C3 16S ribosomal RNA gene, partial sequence	96.92
	**UJA_MA_376**	*Bacillus* sp. (in: Bacteria) strain BA-J-8 16S ribosomal RNA gene, partial sequence	99.72
	**UJA_MA_377**	*Bacillus sp*. DYJL15 16S ribosomal RNA gene, partial sequence	99.44
	**UJA_MA_382**	*Bacillus* sp. (in: Bacteria) strain CPO 4.230 16S ribosomal RNA gene, partial sequence	99.79
	**UJA_MA_383**	*Bacillus* sp. enrichment culture clone SYW15 16S ribosomal RNA gene, partial sequence	99.65
	**UJA_MA_384**	*Bacillus* sp. (in: Bacteria) strain BH40 16S ribosomal RNA gene, partial sequence	99.51
	**UJA_MA_387**	*Bacillus* sp. (in: Bacteria) strain SJU3 16S ribosomal RNA gene, partial sequence	99.72
	**UJA_MA_388**	*Priesta megaterium* strain CGAPGPBBS-034 16S ribosomal RNA gene, partial sequence	99.79
	**UJA_MA_390**	*Priestia aryabhattai* strain H6 16S ribosomal RNA gene, partial sequence	99.72
	**UJA_MA_395**	*Bacillus endophyticus* strain YN14 16S ribosomal RNA gene, partial sequence	99.65
	**UJA_MA_397**	*Priesta megaterium* strain DK2 16S ribosomal RNA gene, partial sequence	99.86
	**UJA_MA_398**	*Peribacillus simplex* strain X10 16S ribosomal RNA gene, partial sequence	99.58
	**UJA_MA_399**	*Peribacillus simplex* strain EGI89 16S ribosomal RNA gene, partial sequence	99.86

	**UJA_MA_403**	Bacterium strain MTL5-77 16S ribosomal RNA gene, partial sequence	97.84
	**UJA_MA_405**	*Priestia megaterium* strain B2 16S ribosomal RNA gene, partial sequence	99.93
	**UJA_MA_406**	*Bacillus thuringiensis* strain L26 16S ribosomal RNA gene, partial sequence	99.79
	**UJA_MA_408**	*Peribacillus simplex* strain P49_BA1H 16S ribosomal RNA gene, partial sequence	99.86
	**UJA_MA_411**	*Brevibacillus choshinensis* strain GNHG-10 16S ribosomal RNA gene, partial sequence	99.86
	**UJA_MA_412**	*Priestia megaterium* strain SX6 16S ribosomal RNA gene, partial sequence	99.86
	**UJA_MA_413**	*Bacillus* sp. T4(2013) 16S ribosomal RNA gene, partial sequence	99.72
	**UJA_MA_416**	*Paenibacillus* sp. strain (5)L 16S ribosomal RNA gene, partial sequence	99.65
	**UJA_MA_417**	*Bacillus velezensis* strain K-13 16S ribosomal RNA gene, partial sequence	99.93

Supplemental figure 1. Antibiotic susceptibility as graphical representation in % of selected culturable olive sporobiota isolates.

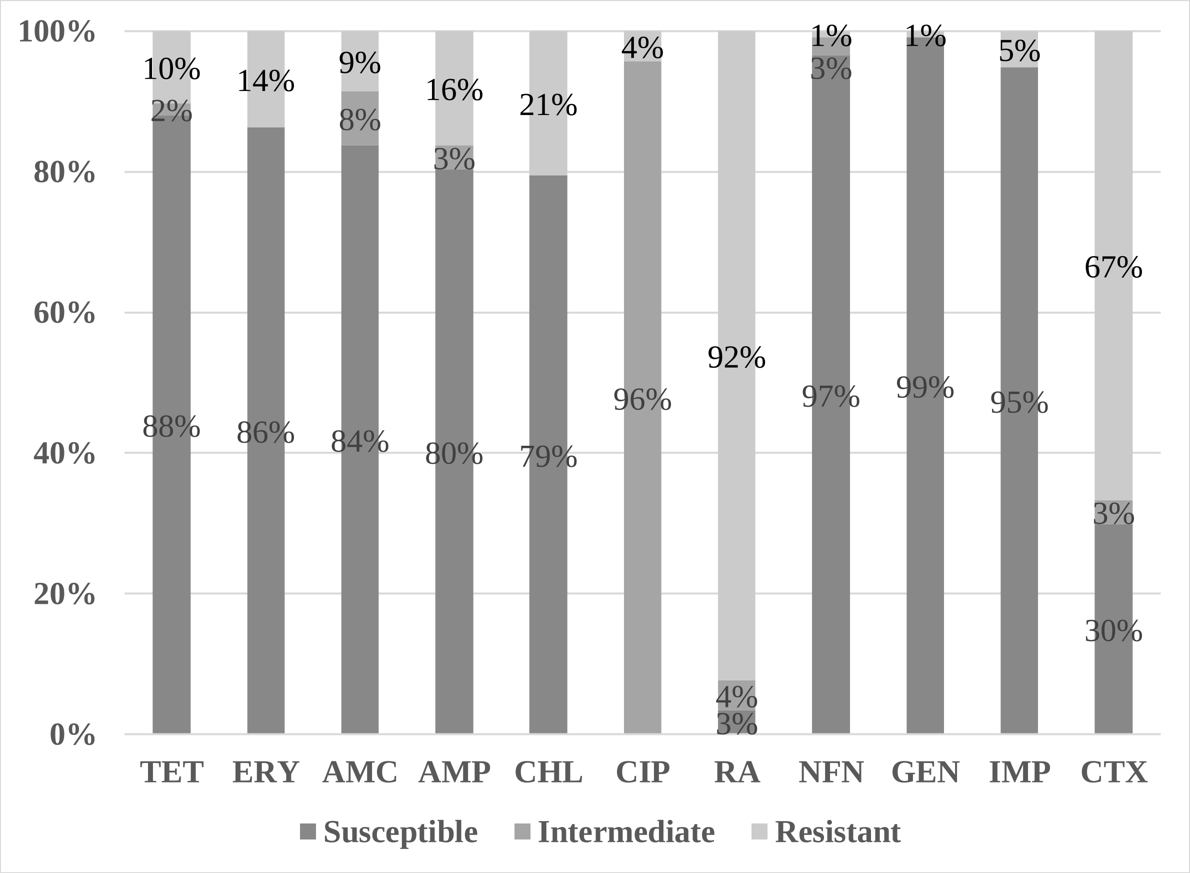

Supplement: Supplemental file 1 — Fig. S1, Table S1. Download spectrum.04013-22-s0001.pdf, PDF file, 0.1 MB [file spectrum.04013-22-s0001.pdf]
